# LPS-induced IL-8 activation in human intestinal epithelial cells is accompanied by specific histone H3 acetylation and methylation changes

**DOI:** 10.1186/1471-2180-10-172

**Published:** 2010-06-14

**Authors:** Tiziana Angrisano, Raffaela Pero, Silvia Peluso, Simona Keller, Silvana Sacchetti, Carmelo B Bruni, Lorenzo Chiariotti, Francesca Lembo

**Affiliations:** 1 Dipartimento di Biologia e Patologia Cellulare e Molecolare "L. Califano", Facoltà di Farmacia e Facoltà di Scienze Biotecnologiche, Università degli Studi di Napoli "Federico II", Via S. Pansini 5, Naples, 80131, Italy; 2 Naples Oncogenomic Center, CEINGE Biotecnologie Avanzate, Università degli Studi di Napoli "Federico II", Via Comunale Margherita 482, Naples, 80145, Italy; 3 Dipartimento di Chimica Farmaceutica e Tossicologica, Facoltà di Farmacia, Università degli Studi di Napoli "Federico II", Via D. Montesano 47, Naples, 80131, Italy

## Abstract

**Background:**

The release of LPS by bacteria stimulates both immune and specific epithelial cell types to release inflammatory mediators. It is known that LPS induces the release of IL-8 by intestinal mucosal cells. Because it is now emerging that bacteria may induce alteration of epigenetic patterns in host cells, we have investigated whether LPS-induced IL-8 activation in human intestinal epithelial cells involves changes of histone modifications and/or DNA methylation at IL-8 gene regulatory region.

**Results:**

RT-PCR analysis showed that IL-8 mRNA levels rapidly increase after exposure of HT-29 cells to LPS. DNA demethylating agents had no effects on IL-8 expression, suggesting that DNA methylation was not involved in IL-8 gene regulation. Consistently we found that 5 CpG sites located around IL-8 transcription start site (-83, -7, +73, +119, +191) were unmethylated on both lower and upper strand either in LPS treated or in untreated HT-29 cells, as well as in normal intestinal mucosa.

Conversely, pretreatment of HT-29 cells with deacetylase inhibitors strengthened the LPS-mediated IL-8 activation. Inhibitors of histone deacetylases could induce IL-8 mRNA expression also in the absence of LPS, suggesting that chromatin modifications could be involved in IL-8 gene regulation. Chromatin immunoprecipitation analyses showed that, concurrently with IL-8 activation, transient specific changes in H3 acetylation and H3K4, H3K9 and H3K27 methylation occurred at IL-8 gene promoter during LPS stimulation. Changes of H3-acetyl, H3K4me2 and H3K9me2 levels occurred early, transiently and corresponded to transcriptional activity, while changes of H3K27me3 levels at IL-8 gene occurred later and were long lasting.

**Conclusion:**

The results showed that specific chromatin modifications occurring at IL-8 gene, including histone H3 acetylation and methylation, mark LPS-mediated IL-8 activation in intestinal epithelial cells while it is unlikely that DNA methylation of IL-8 promoter is directly involved in IL-8 gene regulation in these cells.

## Background

A possible novel additional strategy used by bacterial pathogens during infection is to interfere with host cellular processes by inducing epigenetic modifications and, consequently, determining a new specific cell transcriptional profile. Bacteria or their components could be a stimulus to change the genetic program of the target cells through epigenetic mechanisms [1,2]. These mechanisms may operate at gene-specific level and include both chromatin modifications, orchestrated by chromatin-remodeling complexes and histone-modifying enzymes, and DNA methylation, directed by DNA-methyltransferases. Histone acetylation is in general associated to an active state of the chromatin while the effects of histone methylation may be associated with either transcriptional activation or repression, depending on which lysyl residue is modified [3,4] and whether this residue is mono, di or trimethylated. Among the best studied H3 lysine modifications are di- and trimethylation of H3 on lysine 9 and lysine 27 (H3K9me2 and H3K27me3), associated with closed chromatin, and dimethylation of H3 on lysine 4 (H3K4me2) that marks active chromatin state. DNA methylation of CpG sites at gene regulatory regions is in general related to transcriptional repression and it is believed to be a more stable epigenetic mark compared to histone modifications [5,6]. However, chromatin modifications and DNA methylation are strictly linked and can associate or interfere with each other [5,7].

Bacterial-host interactions have been shown to affect the histone acetylation, phosphorylation and methylation state at the TLR4 and IL-8 promoter in host cells [8-10].

The effects of lipopolysaccharide (LPS) on some aspects of host epigenetics have been recently reported in macrophages and T lymphocytes. In T lymphocytes, LPS stimulation of TLR4 induces histone acetylation and H3S10 phosphorylation allowing for NF-κB to gain access to the IL-12 promoter [11,12]. Moreover LPS-tolerance, associated with immunosuppression and poor prognosis [13], has been shown to be controlled by epigenetic changes including methylation of H3K9 [14-16]. LPS is the major component of the outer membrane of gram negative bacteria. The release of LPS by bacteria stimulates both immune and specific epithelial cell types to release inflammatory mediators. Although the effects of LPS have been deeply studied on macrophages and T-cells, only few studies addressed the LPS effects on the intestinal epithelial cells [17,18]. This is of particular importance because the intestinal epithelial cells represent a key component of the mucosal immune system and are able to express inflammatory genes in response to LPS [17,18]. These studies addressed the signaling pathways leading to LPS responsiveness of HT-29 cells, a human intestinal epithelial cell line, and demonstrated that LPS response is mediated by gamma interferon (IFN-γ) that induces the expression of the Toll-like receptor 4-MD-2 complex [18]. As a result of LPS stimulation, the proinflammatory cytokine IL-8 accumulates in the culture medium of HT-29 cells.

In this work we have investigated whether epigenetic mechanisms are involved in LPS induced IL-8 gene activation in human intestinal epithelial cells. We found that both histone acetylation and methylation changes at IL-8 promoter, but not DNA methylation, are involved in IL-8 gene activation upon LPS induction.

## Results and Discussion

### Kinetics of LPS-mediated IL-8 gene activation in HT-29 cells

**Figure 1:**
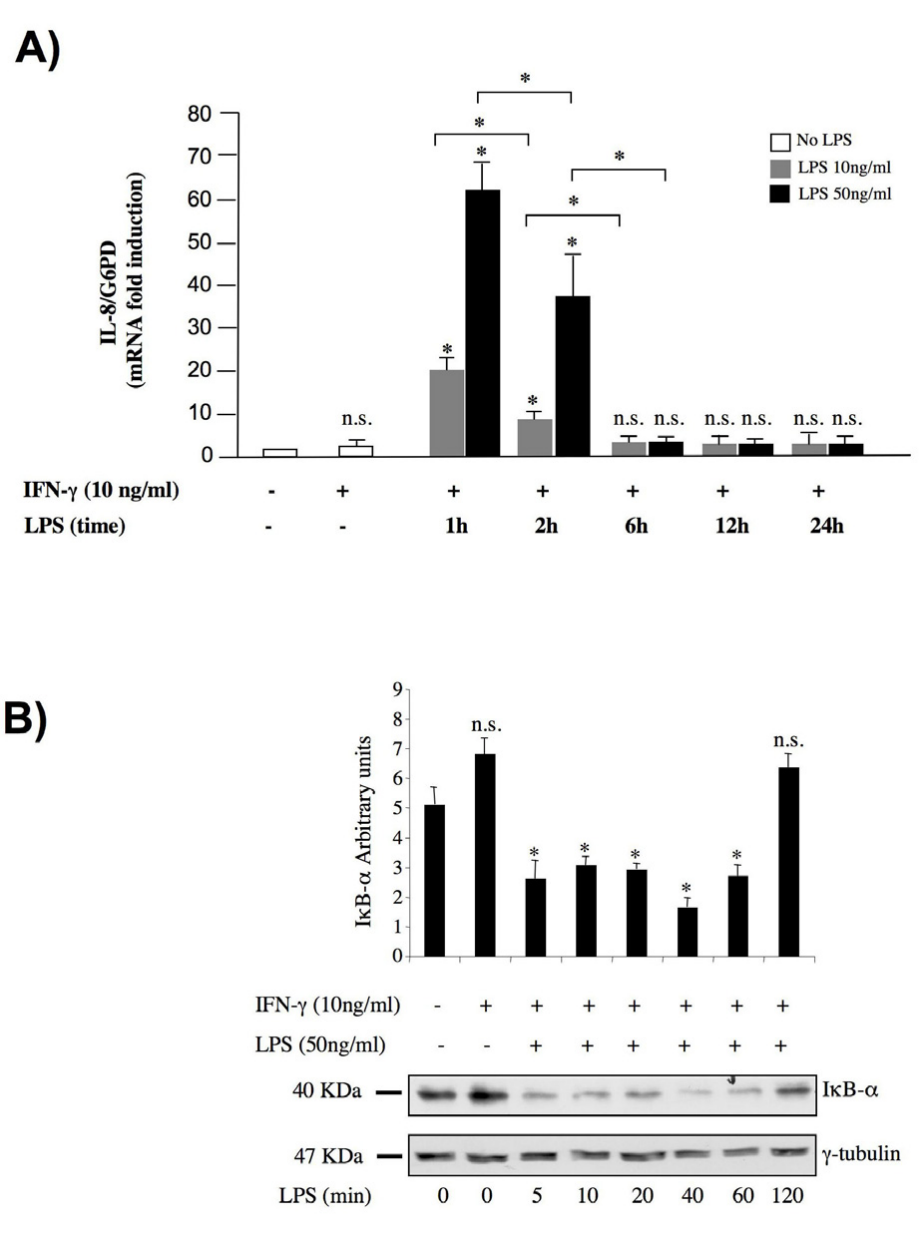
**Time course analysis of LPS-mediated IL-8 gene activation**. (A) Total RNA was isolated at indicated time points after LPS administration and used in real-time PCR reactions. Where indicated, HT-29 cells were pre-treated with IFN-γ (10 ng/ml) for 24 hours. The IL-8 mRNA levels were normalized to G6PD levels and expressed as relative to untreated control cells. Data points represent the average of triplicate determinations ± SD. Similar results were obtained in 3 independent experiments. *, *p *< 0.01; n.s.= not significant, in comparison to control culture without IFN-γ. (B) Lysates were collected at the indicated time points in RIPA buffer and 50 μg of protein samples were loaded for electrophoresis. The expression levels of IκB-α were detected using anti-IκB-α antibodies. The levels of γ-tubulin were used to demonstrate equal loading. Protein levels were quantified using the software Quantity One (Bio-Rad). The IκB-α protein levels were normalized to γ-tubulin levels and expressed as relative to untreated control cells. Data points represent the average of three independent experiments ± SD. A representative blot is shown. *, *p *< 0.01.

HT-29 cells are responsive to LPS and IL-8 protein accumulates in the culture medium upon such treatment [18]. We performed a time course analysis of IL-8 mRNA expression upon LPS stimulation. HT-29 cells were primed with IFN-γ (see Methods) in order to allow myeloid differentiation protein 2 (MD-2) expression, which is required for HT-29 LPS responsiveness as previously described [18]. Activation of MD-2 expression upon IFN-γ treatment was confirmed in HT-29 cells used in this study by semiquantitative RT-PCR analysis (data not shown). Then the primed HT-29 cells were treated with LPS (10 and 50 ng/ml) and IL-8 mRNA levels were measured by real time PCR at different time points (Figure 1A). IL-8 mRNA expression showed a striking increase in response to LPS, reaching a maximum 1 hour after stimulation with 50 ng/ml LPS and gradually decreasing at later times. These results were confirmed by semiquantitative RT-PCR analysis (data not shown).

Because NF-κB has a critical role in LPS-mediated gene activation [17,19], we measured by western blot analysis the protein levels of the NF-κB inhibitor IκB-α at short intervals after LPS treatment. Results shown in Figure 1B demonstrate that IκB-α was rapidly degraded in HT-29 cells upon LPS stimulation. A significant decrease in IκB-α was already observed 5 minutes after stimulation and persisted up to 60 minutes. These data are consistent with the observed kinetics of IL-8 mRNA expression (Figure 1A).

### Inhibitors of histone deacetylases but not of DNA methyltransferases reactivate IL-8 gene expression in HT29 cells

**Figure 2:**
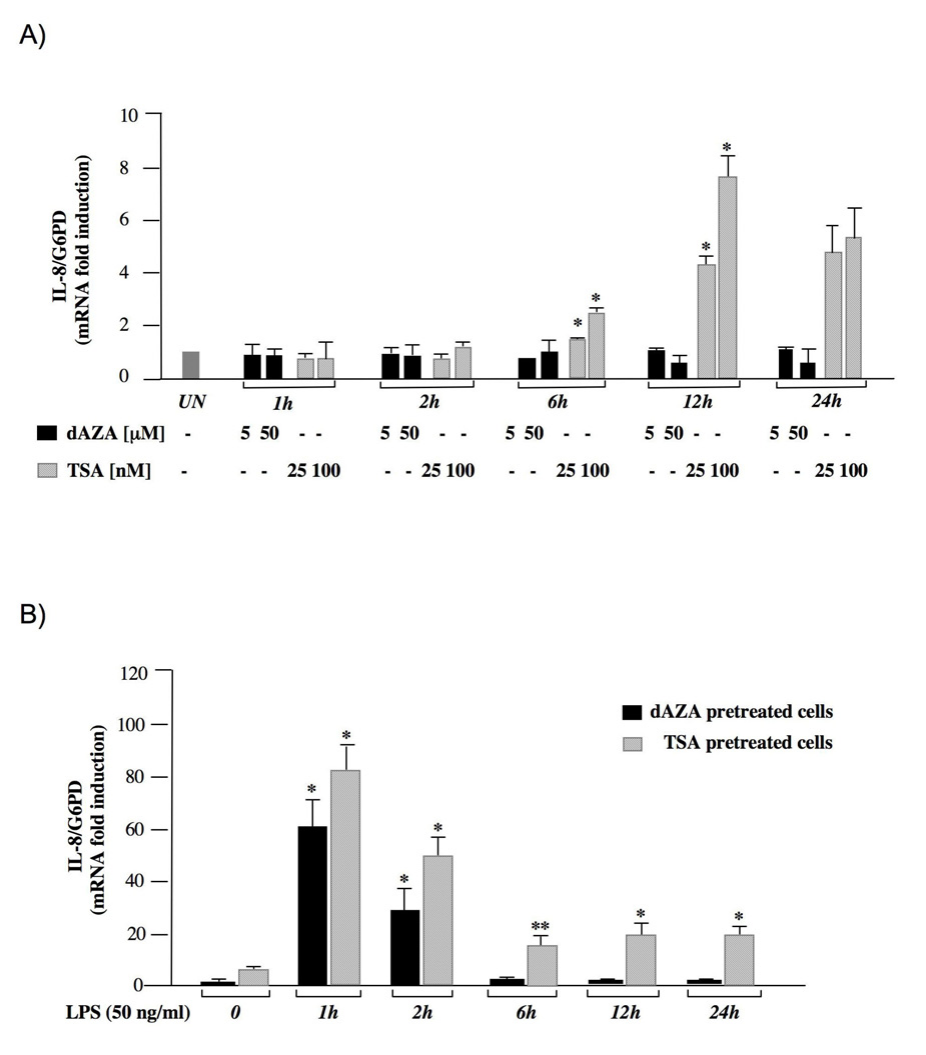
**Effects of TSA and 5-dAZA on IL-8 gene expression**. (A) HT-29 cells were treated with the indicated concentration of drugs for the indicated times and total RNA was analyzed by real-time PCR. (B) HT-29 cells were pre-treated with 5-dAZA (50 μM) (black bars) or TSA (100 nM) (striped bars) for 24 hours (control) and then treated with LPS (50 ng/ml) and total RNA was extracted at the indicated time points after LPS administration and subjected to real time PCR analysis. The IL-8 mRNA levels were normalized to G6PD levels and expressed as relative to untreated control cells. Data points represent the average of triplicate determinations ± SD. Similar results were obtained in 3 independent experiments. Statistical analyses were performed compared to respective untreated control cells. *, *p *< 0.01; **, *p *< 0.05; absence of asterisks = not significant.

In order to investigate whether IL-8 gene may be regulated by DNA methylation or histone acetylation state, we treated HT-29 cells with trichostatin (TSA), an inhibitor of histone deacetylases and with 5-deoxy-azacytidine (5-dAZA), a drug that inhibits DNA methyltransferases. RT-PCR experiments were then performed to measure IL-8 mRNA levels at different times after drug induction. Results shown in Figure 2A indicated that TSA treatment induces a concentration-dependent increase of IL-8 mRNA levels starting after 6 hours. The observed changes in IL-8 gene expression were similar both in primed and in unprimed cells (data not shown), indicating that TSA can induce expression of IL-8 independently from the IFN-γ pathway. Conversely, no effects were observed when HT-29 cells were treated with 5 μM or 50 μM 5-dAZA (Figure 2A).

Then we examined the effects of TSA and 5-dAZA on LPS induced IL-8 expression (Figure 2B). HT-29 cells were primed with IFN-γ, pretreated for 24 hours with TSA or 5-dAZA, and then the cells were stimulated with 50 ng/ml LPS. We found that cells pretreated with 5-dAZA showed an IL-8 activation pattern very similar (*p *= n.s.) to that observed in cells treated with LPS alone (Figure 1A), while TSA pretreatment significantly enhanced the LPS-mediated IL-8 activation (*p *< 0.05). Taken together these data suggest that histone acetylation state but not DNA methylation may influence IL-8 expression in intestinal derived HT-29 cells.

### DNA methylation analysis of IL-8 promoter region

**Figure 3:**
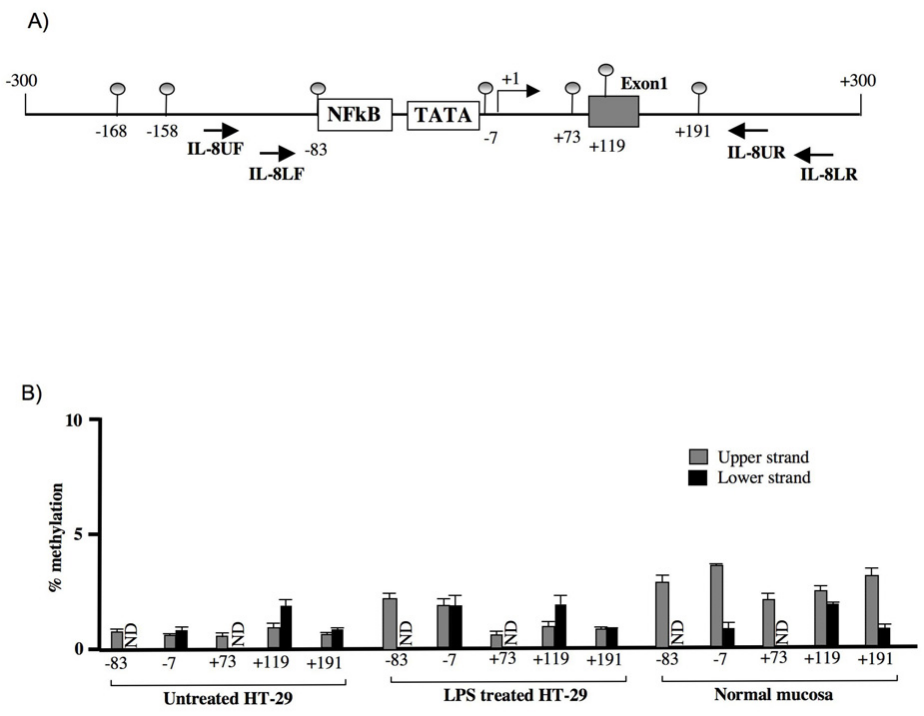
**Methylation status of individual CpG sites on upper and lower strands at IL-8 promoter**. (A) Schematic representation of human IL-8 gene promoter. Regulatory upstream region (proximal NF-κB binding site and TATA box), Transcriptional start site (arrow) and exon 1 (gray box) are indicated. The relative positions of each CpG site present in the analyzed region and of the primers utilized for amplification are indicated. (B) Methylation degree at CpG sites -83, -7, +73, +119, and +191 on both upper (gray bars) and lower strand (black bars) was measured in untreated HT-29, in cells treated 24 hours with LPS and in normal colon mucosa samples by MALDI-TOF analysis. Methylation of sites -83 and -73 on lower strand could not be determined by MALDI analysis (ND). Each experiment was repeated three times on three different samples. Shown are the average values for each indicated CpG site ± SD.

Because the DNA methylation state at promoter regions may indeed influence the chromatin changes during gene activation, we sought to validate HT-29 cells as a good model to study chromatin modification at IL-8 locus. First, in order to confirm that DNA methylation is not involved in IL-8 gene regulation in HT-29 cells, we analyzed the methylation state of 5 CpG sites lying around the IL-8 gene transcription start site (-83, -7, +73, +119, +191), both on upper and lower strands by MALDI-TOF analysis of genomic DNA extracted from untreated or LPS-treated cells (Figure 3A). We found that all five sites were completely unmethylated (0-2%) both in untreated and in LPS-treated cells (Figure 3B). Then, in order to investigate whether the observed DNA methylation profiles at the IL-8 locus were a specific feature of HT-29 cell line or resembled those present in human tissues, we analyzed DNA from normal colon mucosa samples. Results showed that, similarly to HT-29 cells, CpG methylation at IL-8 locus in normal colon mucosa displayed an almost unmethylated state (0-4%) on both upper and lower strands (Figure 3B), confirming that HT-29 cells may be used to study chromatin dynamics at IL-8 gene. Interestingly, previous studies addressing the methylation state at IL-8 gene in several breast cancer cell lines, showed that the CpG sites located at the IL-8 promoter region were unmethylated in both metastatic and non-metastatic cell lines [20].

### LPS-mediated IL-8 gene activation is accompanied by both histone H3 acetylation and methylation changes

**Figure 4:**
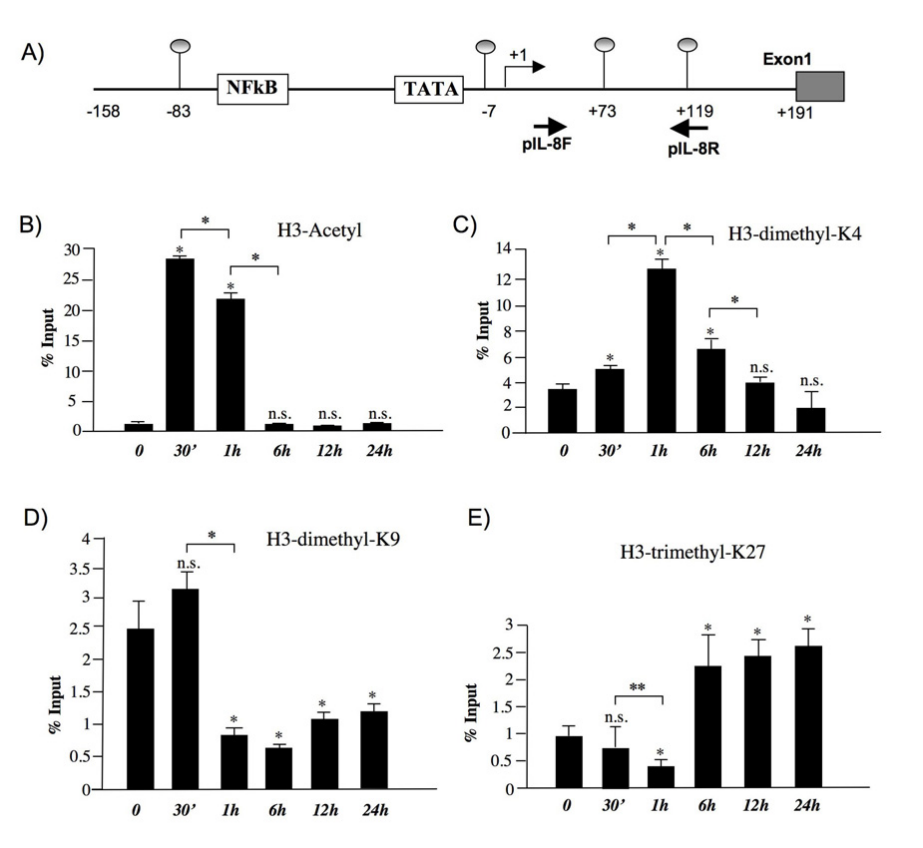
**LPS induces early histone H3 methylation and acetylation changes at the promoter region of IL-8 gene**. Chromatin from HT-29 cells was harvested at the indicated time points after LPS exposure. (A) Schematic representation of IL-8 promoter region, as in Figure 3. Positions of the primers used for ChIP analyses are shown. Presented are the results of ChIP analyses using anti-acetyl-H3 (B) anti-dimethyl-H3K4 (C), anti-dimethyl-H3K9 (D) and anti-trimethyl-K27 (E) antibodies. DNA sequences recovered after the indicated times of LPS treatment were quantified by real-time PCR using the primers indicated above. Average% input ± S.D from 4 independent experiments are plotted. *, *p *< 0.01; **, *p *< 0.05; n.s.= not significant, compared to control cells.

Then we performed chromatin immunoprecipitation (ChIP) experiments in order to investigate whether specific changes in histone modifications occurred at IL-8 promoter during LPS-induced gene activation. First we determined whether IL-8 activation corresponded to increased levels of histones H3 acetylation in the promoter region of IL-8 gene. Cells were incubated with LPS for different times and chromatin was immunoprecipitated with anti acetyl-H3 antibodies; then PCR amplifications were performed using promoter-specific primers (see Figure 4A and Methods section). We found that upon LPS treatment H3 acetylation state was transiently modulated. The histone H3 was highly acetylated after 30 minutes while the deacetylated state was restored after 6 hours (Figure 4B). Hyper-acetylation of histone H3 is in agreement with expression pattern of the IL-8 gene. Then, we determined whether the induction of IL-8 gene was accompanied by modifications of histone methylation state. Antibodies against dimethylated H3K4 (H3K4me2), dimethylated H3K9 (H3K9me2) and trimethylated H3K27 (H3K27me3), were used in ChIP assays. We found that the levels of H3K4me2 were low in untreated HT-29 cells, significantly increased 1 hour after LPS administration, and gradually returned to basal levels within 24 hours (Figure 4C). Conversely, H3K9me2 showed a significant increase after 30 minutes and then rapidly decreased at 1 hour remaining lower than basal levels after 24 hours (Figure 4D). These results, examined together with the expression data (see Figure 1), are in agreement with the repressive role of H3K9me2 and with the activating role described for H3K4me2 in gene transcription [3,4,7]. The sharp increase in H3K9me2 levels observed at 30 minutes time point at IL-8 promoter, despite the transcriptional activated status, could be explained by a possible concomitant demethylation of trimethylated H3K9 and consequent transient accumulation of the dimethylated form. However, already after 1 hour, the H3K9me2 demethylation was clearly evidenced by a significant reduction of its levels that remained lower than basal levels at IL-8 promoter 24 hours after LPS stimulation.

Very interestingly, H3K27me3 levels were initially very low but then increased substantially starting at 6 hours and remained high 24 hours after LPS stimulation (Figure 4E).

H3K27 trimethylation is catalyzed by Polycomb group (PcG) protein complexes [21,22], which have been shown to be involved in cytokines genes reprogramming occurring in both epithelial and macrophage cells in response to bacterial products and inflammation-related stimuli [23,24]. It is also worth to note that when all the above ChIP assays were performed in unprimed HT-29 cells (not pre-treated with interferon-γ) we did not detect significant changes in histone H3 methylation state during the same time course (data not shown) suggesting that the observed chromatin modifications are dependent on the MD2/TLR4 pathway. However, because it is well known that even "pure" LPS preparations may be contaminated with lipoproteins, we cannot definitively exclude that the observed chromatin modifications could be influenced by TLR2 signaling.

Taken together our data indicate that while changes of H3-acetyl, H3K4me2 and H3K9me2 state in the IL-8 promoter region occur rapidly, transiently and correspond to transcription activation, the changes of H3K27me3 levels occur at a later time and are long lasting.

Finally it should be considered that a strong mark of gene repression, such as H3K27me3, could predispose to a more repressed state of IL-8 gene and, thus, could render the gene less responsive to further LPS stimulation. Moreover, H3K27me3 is also related to DNA hypermethylation that has been shown to occur in intestinal cancer at PcG target genes. In particular, it has been recently demonstrated that hypermethylation of PcG target genes in intestinal cancer is mediated by inflammation [24]. Thus, although our data indicate that DNA methylation is not directly involved in LPS response, such phenomenon may occur later, after prolonged exposure to LPS, as a consequence of PcG proteins recruitment at IL-8 gene.

In the near future it will be very important to investigate whether the observed increase in H3K27me3 levels at IL-8 gene in response to LPS may occur in intestinal mucosa and may represent a stable epigenetic mark; in this case the observed modifications could be potentially involved in two important phenomena such as the LPS tolerance, and the hypermethylation of PcG target genes in intestinal cancer.

## Conclusions

Our data demonstrate an important role of histone modifications, including histone H3 acetylation and H3K4, H3K9 and H3K27 methylation state, in LPS-mediated IL-8 gene activation in intestinal epithelial cells. In particular we demonstrate that H3-acetyl, H3K4me2 and H3K9me2 changes are early, transient and correlate with the modulation of IL-8 transcriptional activity. Conversely, increase of H3K27me3 levels at IL-8 gene occurs later and is long lasting. Our data provide novel insights into the epigenetic mechanisms that control transcription and gene expression in LPS response.

## Methods

### Cell culture

The human colon cell lines HT-29 were grown in Dulbecco's Modified Eagle's Medium supplemented with 10% fetal bovine serum (Life Technologies), 2 mM glutamine, penicillin (25 U/mL) and streptomycin (25 μg/mL) in a 5% CO_2 _atmosphere at 37°C. Cells were pretreated with Human interferon-γ (INF-γ) (Roche Applied Science, Germany) 10 ng/ml for 12 hours or control medium, washed, and then stimulated with LPS 50 ng/ml. LPS (*Escherichia coli*, O55:B5) were purchased from Sigma-Aldrich (St. Louis, MO) and reconstituted in endotoxin-free water. 5-aza-2-deoxyazacytidine (ICN Biomedical Inc.) treatments were performed at 5 μM and 50 μM final concentration while trichostatin (TSA) (Sigma Aldrich) was used at 25 and 100 nM.

### Western Blot Analysis

Cell extracts were prepared in Nonidet P40 lysis buffer with 1 mM PMSF and Complete™ protease inhibitors mix (Roche, Indianapolis, IN, USA). 50 μg of proteins were resolved by electrophoresis using 10% SDS-PAGE gels and transferred to BA 85 0.45 μm PROTAN nitrocellulose filters (Schleicher & Schnell, Inc., Dassel, Germany). The blots were incubated with rabbit anti-IκB-α antibodies (Santa Cruz Biotechnology, Santa Cruz, CA, USA) and mouse anti-γ-tubulin antibodies (Sigma-Aldrich Corp. St. Louis, MO, USA) as a control for protein loading. Immunoblots were stained with correspondent secondary antibodies IgG (Amersham Pharmacia Biotech, Buckinghamshire, UK), and revealed with the enhanced chemiluminescence detection system IgG (ECL, Amersham Pharmacia Biotech, Buckinghamshire, UK). Western blot analyses of each sample were performed more than three times. Protein levels were quantified using the software Quantity One (Bio-Rad).

### Quantitative and semiquantitative RT-PCR analysis

Total RNA was isolated with RNeasy extraction kit QIAGEN (Qiagen,GmBh) according to the manufacturer instructions. The integrity of the RNA was assessed by denaturing agarose gel electrophoresis (presence of sharp 28S, 18S and 5S bands) and spectrophotometry. To ensure that RNA samples were not contaminated by DNA, negative controls were obtained by performing the PCR on samples that were not reversed-transcribed but otherwise identically processed. 1 μg of total RNA of each sample was reverse-transcribed with QuantiTect^® ^Reverse Transcription (Qiagen) using an optimized blend of oligo-dT and random primers according to the manufacturer's instructions. Quantitative PCR amplifications were performed using QuantiTect SYBR Green (Qiagen) in a Chromo4 Real Time thermocycler (BIORAD). Following primers were used for IL-8 cDNA amplification: cIL-8F (forward) 5'-ggcacaaactttcagagacag-3' and cIL-8R (reverse) 5'-acacagagctgcagaaatcagg-3'; G6PD gene was used as housekeeping gene for PCR reaction: G6F (forward) 5'-acagagtgagcccttcttcaa-3' and G6R (reverse) 5'-ggaggctgcatcatcgtact-3'. The quantitative PCR conditions were: 95°C for 15 minutes followed by 40 cycles of 95°C for 15 seconds, 60°C for 30 seconds, and 72°C for 30 seconds. Calculations of relative expression levels were performed using the 2^-ΔΔCt ^method [25] and take into account the values of at least three independent experiments. Semiquantitative PCR reactions were performed for the assessment of IL-8 expression, using cIL-8F and cIL-8R primers, and MD-2 expression using the following primers: MDF (forward) 5'-ggctcccagaaatagcttcaac-3' and MDR (reverse), 5'-ttccaccctgttttcttccata-3'; GAPDH was used as a housekeeping gene for normalization using the following primers: GAPF (forward) 5'-ggtcgtattgggcgcctggtcacc-3' and GAPR (reverse) 5'- cacacccatgacgaacatgggggc-3'. Each reaction was performed in triplicate. The conditions used for semiquantitative PCR were 1 minute at 94°C, 1 minute at 60°C and then 2 minutes at 68°C for 30 cycles. The PCR products were separated on a 1.5% agarose gel and stained with ethidium bromide.

### DNA methylation analysis

Genomic DNA was isolated from cultured cells and from tissue samples using DNeasy Blood and Tissue extraction kit (Qiagen) according to the manufacturer's instructions. Colon samples were obtained from the tissue bank of the Naples Oncogenomic Center (NOGEC). Normal mucosa samples were taken from macroscopically and microscopically unaffected areas of a colon cancer specimen. Sodium bisulfite conversion of 1 μg of genomic DNA was performed using EZ DNA Methylation Kit (Zymo Research). DNA methylation analysis was performed using the SEQUENOM MassARRAY platform. This system utilizes MALDI-TOF mass spectrometry in combination with RNA base specific cleavage (MassCLEAVE). A detectable pattern is then analyzed for methylation status. PCR primers to analyze IL-8 promoter region, designed by using Epidesigner http://www.epidesigner.com, were: for upper strand region (-137 to +246) IL-8UF 5'-aggaagagagGGAAGTGTGATGATTTAGGTTTGTT-3' and IL-8UR 5' cagtaatacgactcactatagggagaaggctCCAAAACATCAAAAATAACTTTACTATCT-3'; for lower strand (region -113 to +264) IL-8LF 5'- aggaagagagAAAAAGGATGTTTGTTATTAAAGTATTAAG-3' and IL-8LR 5'- cagtaatacgactcactatagggagaaggctCCCTAAAAAAATAAACCATCAATTAC-3'. For reverse primer, an additional T7 promoter tag for in vivo transcription was added, as well as a 10-mer tag on the forward primer to adjust for melting-temperature differences. The sensitivity of methylation assay was evaluated using Universal methylated and unmethylated Human DNA Standards (Zymo Research Corporation, Orange USA) and the standard error was found to be ± 3%. The MassCLEAVE biochemistry was performed as previously described [26]. Mass spectra were acquired by using a MassARRAY Compact MALDI-TOF (Sequenom) and spectra's methylation ratios were generated by the Epityper software v1.0 (Sequenom). The whole procedure was performed at Sequenom GmbH Laboratories (Hamburg, Germany).

### Quantitative ChIP analysis

Cells were plated at a density of ~ 3-5 10^6 ^in 100 mm Petri dish 24 h before the treatments. Cells were cross-linked by adding 1% formaldehyde for 15 minutes at room temperature in shaking. Glycine was added to a final concentration of 125 mM for 5 minutes at room temperature in shaking. Cells were rinsed twice with cold PBS supplemented with 500 μM PMSF and harvested in five pellet-volumes of Cell Lysis Buffer (5 mM PIPES pH 8.0, 85 mM KCl, 0.5% NP40) supplemented with 1 mM PMSF and Complete™ protease inhibitors mix. Lysates were incubated for 30 minutes at 4°C and then passed through ten dounce cycles. They were subsequently centrifuged and nuclei were collected. Nuclei were then resuspended in 250 μL Sonication Buffer (0.3% SDS, 10 mM EDTA, 50 mM Tris-HCl ph 8.0) supplemented with 1 mM PMSF and Complete™ protease inhibitors mix and incubated for 60 minutes at 4°C. Chromatin was sonicated to an average DNA length of 300-800 bp using a 3 mm (small size) tip equipped Bandelin Sonoplus UW-2070 sonicator with 5 × 10 seconds cycles of pulses (specific cycle 0.3, Power 30%) alternated by 60 seconds of rest. Sonicated samples were centrifuged and the supernatant was collected. 80 μg of chromatin were diluted with Dilution Buffer (0.01% SDS, 1.2 mM EDTA, 16.7 mM Tris-HCl pH 8.0, 1,1% TRITON X-100, 167 mM NaCl), precleared (2 hours) by incubation with 20 μL Salmon Sperm DNA/Protein A Agarose-50% Slurry (Upstate Biotechonology, Dundee; UK) and subjected to immunoprecipitation with specific antibodies with rotation over-night at 4°C. Antibodies used for ChIP assays were: anti-H3Ac, anti di-methyl-H3K9, anti tri-methyl-H3K27 (Upstate Biotechnology) and anti-di-methyl-H3K4 (Abcam Inc.). Immunocomplexes were collected by adsorption onto 30 μL Salmon Sperm DNA/Protein A Agarose-50% Slurry and the beads were washed (four times) sequentially with Low Salt Washing Buffer (0.1% SDS, 2 mM EDTA, 20 mM Tris-HCl pH 8.0, 1% Triton X-100, 150 mM NaCl), High Salt Washing Buffer (0.1% SDS, 2 mM EDTA, 20 mM Tris-HCl pH 8.0, 1% Triton X-100, 500 mM NaCl) and LiCl Washing Buffer (Upstate). Precipitates were washed with TE Buffer (10 mM Tris-HCl pH 8.0 and 1 mM EDTA), and antibody-chromatin fragments were eluted from the beads with 1% sodium dodecyl sulphate in 0.1 M NaCO_3_. Cross-links were reverted by adding 200 mM NaCl and heating at 65°C overnight. 40 mg/mL RNase A and 20 mg/mL proteinase K, 10 mM EDTA and 40 mM Tris-HCl pH 6.5 were added and samples were then incubated 2 hours at 45°C. Samples were then extracted in phenol-chloroform-isoamylic acid (25:24:1), ethanol-precipitated and finally centrifuged at 13000 rpm for 45 minutes at 4°C. Pellets were washed with 70% ethanol, centrifuged at 8000 rpm for 5 minutes at 4°C and finally resuspended in 60 μL of H_2_O. 2 μL of each sample were used as template for subsequent PCR analysis and 32 amplification cycles were used. Amplification of the IL-8 promoter fragment, using SYBR^®^Green Taq, was performed using the primers: pIL-8F (forward) 5'- CAGAGACAGCAGAGCACAC-3' and pIL-8R (reverse) 5'-ACGGCCAGCTTGGAAGTC-3' amplifying a 101 bp fragment. All PCR signals from immunoprecipitated DNA were normalized to PCR signals from non-immunoprecipitated input DNA. The signals obtained by precipitation with the control IgG were subtracted from the signals obtained with the specific antibodies. Results are expressed as percentage of the input: signals obtained from the ChIPs were divided by signals obtained from an input sample; this input sample represents the amount of chromatin used in the ChIP. Calculations take into account the values of at least three independent experiments.

### Statistical Analysis

Statistical significance between groups was assessed by Student's *t *test. Data are expressed as means ± standard deviation (SD). All experiments were repeated at least three times. A *p *value < 0.05 was considered to be statistically significant.

## Abbreviations

LPS: lipopolysaccharide; IL-8: interleukin-8; RT-PCR: reverse-transcription-polymerase chain reaction; TLR4: Toll-like receptor 4; NF-κB: nuclear factor-κB; TSA: trichostatin; 5-dAZA: 5'-deoxy-azacytidine; IFN-γ: interferon-gamma; ChIP: chromatin immunoprecipitation; MD-2: myeloid differentiation protein 2; PcG: Polycomb protein complexes; IκBs: inhibitors of NF-κB.

## Authors' contributions

TA carried out the chromatin and DNA methylation analysis. RP carried out the gene expression analysis and immunoassays. SP participated in the chromatin immunoprecipitation assays. SK participated in the DNA methylation analysis and in the interpretation of data. SS performed statistical analysis and participated in the DNA methylation analysis. CBB participated in the design and coordination of the study. LC participated in the design and coordination of the study and drafted the manuscript. FL conceived of the study and participated in its design and coordination. All authors read and approved the final manuscript.
